# Activated PyK2 and Its Associated Molecules Transduce Cellular Signaling from the Cancerous Milieu for Cancer Metastasis

**DOI:** 10.3390/ijms232415475

**Published:** 2022-12-07

**Authors:** Dongun Lee, Jeong-Hee Hong

**Affiliations:** Department of Health Sciences and Technology, Lee Gil Ya Cancer and Diabetes Institute, Gachon Advanced Institute for Health Sciences & Technology, Gachon University, 155 Getbeolro, Yeonsu-gu, Incheon 21999, Republic of Korea

**Keywords:** PyK2, migration, metastasis, acidic milieu, PyK2-interactive proteins

## Abstract

PyK2 is a member of the proline-rich tyrosine kinase and focal adhesion kinase families and is ubiquitously expressed. PyK2 is mainly activated by stimuli, such as activated Src kinases and intracellular acidic pH. The mechanism of PyK2 activation in cancer cells has been addressed extensively. The up-regulation of PyK2 through overexpression and enhanced phosphorylation is a key feature of tumorigenesis and cancer migration. In this review, we summarized the cancer milieu, including acidification and cancer-associated molecules, such as chemical reagents, interactive proteins, chemokine-related molecules, calcium channels/transporters, and oxidative molecules that affect the fate of PyK2. The inhibition of PyK2 leads to a beneficial strategy to attenuate cancer cell development, including metastasis. Thus, we highlighted the effect of PyK2 on various cancer cell types and the distribution of molecules that affect PyK2 activation. In particular, we underlined the relationship between PyK2 and cancer metastasis and its potential to treat cancer cells.

## 1. Introduction

### 1.1. General Pathway of PyK2 Phosphorylation

**Figure 1:**
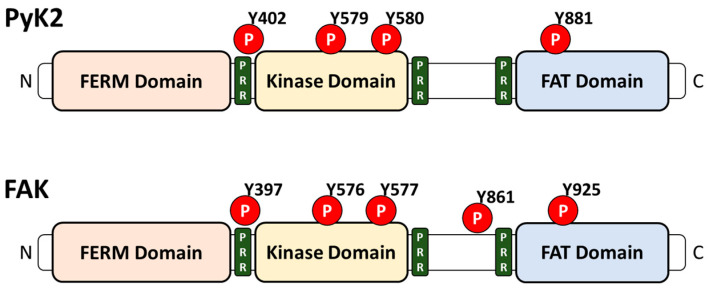
Schematic structure of PyK2 and FAK. Similar structures of PyK2 and FAK, both of which contain a FERM domain, kinase domain, and FAT domain, from the N-terminal (N) to the C-terminal (C). PyK2 and FAK contain a proline-rich region (PRR) and a phosphorylation site (P).

Proline-rich tyrosine kinase 2 (PyK2) is a member of the proline-rich cytoplasmic tyrosine kinase family, which is ubiquitously expressed and dominantly localized in neuronal cells, endothelial cells, and hematopoietic cells [1,2,3,4,5]. PyK2 is phosphorylated by the initiation of extracellular signals during recruitment to the perinuclear membrane or nucleus [6]. Activated PyK2 phosphorylates the tyrosine residues of the target proteins. The PyK2 has a FERM (F for 4.1 protein, E for ezrin, R for radixin, and M for moesin) domain, which is a regulating domain in the N-terminus, and a focal adhesion targeting (FAT) domain located in the C-terminus (Figure 1) [7]. As a focal adhesion kinase, PyK2 transduces extracellular signals and coordinates cellular adhesion and cytoskeletal dynamics to regulate cell migration, proliferation, and survival [8]. PyK2 has a critical role in various cellular mechanisms, such as the migration of immune cells, including lymphocytes, macrophages [9,10], and glioma cells [11]. PyK2 also regulates the adhesion of T cells [12] and lipopolysaccharide (LPS)-induced IL-8 production in human endothelial cells [13].

In addition to PyK2, tyrosine kinases have two other members, focal adhesion kinase (FAK) and Src [14]. The structure of FAK shares a 65% similarity with the structure of PyK2 and contains the same three domains, including FERM, a central catalytic kinase domain, and a FAT domain (Figure 1) [3,5,15,16]. Although the structures of FAK and PyK2 are similar, they play distinct roles. The deletion of the FAK gene impaired mesodermal development, but the loss of PyK2 did not induce developmental impairments in mice [9,10,17]. While FAK is ubiquitous, PyK2 has limited expression [16]. PyK2 compensates for FAK expression. The deletion of FAK increased the expression of PyK2 in a mouse model [18,19]. Src is composed of family members that include Src, Lyn, Fyn, Yes, Hck, Fgr, Blk, and Lck [20]. The activity of Src has been studied in human lung, breast, colon, pancreatic, and gastric cancer [21,22,23,24,25]. Although the Y402 phosphorylation site of PyK2 is auto-phosphorylated [15], Src, another tyrosine kinase, is essential to the subsequent phosphorylation of PyK2 at other sites, including Y579, Y580, and Y881 [26,27,28]. In addition, Src has the SH2 domain which binds to PyK2 phosphorylation sites [7]. Especially in macrophages, LPS-stimulated IL-10 production is required to form a PyK2-Src complex with Src homology region 2 domain-containing phosphatase (SHP)-1 [29]. Although SHP-1 indirectly binds Src and directly binds PyK2, the inhibition of SHP-1 expression reduced the phosphorylation of Src and PyK2 [29]. Additionally, in SHP-1 knockout mice, macrophages showed the aberrant production of IL-10 after LPS stimulation [29]. Although the relationship between PyK2 and other kinases in the immune system has been extensively addressed, the roles of PyK2 in cancer progression, including the homeostasis of pH and intracellular Ca^2+^ signaling, have not been revealed. Thus, we elucidated the modulatory role of PyK2 and PyK2-associated molecular mechanisms in a cancer system.

### 1.2. PyK2 and Cancer-Favorable Acidified Milieu

The acidic pH of the extracellular milieu, which ranges from 6.4 to 7.0 [30], is a key feature of the cancer environment [31]. Extracellular acidosis induces numerous functions involved in cancer metabolism, especially cancer metastasis [31]. To develop metastasis, cancer cells proceed with the sequential steps of proliferation, epithelial-to-mesenchymal transition (EMT), invasion, transport, colonization, and angiogenesis [32]. Acidification of the cancer extracellular matrix induces EMT and invasion. For EMT, the loss of cell-to-cell adhesion and remodeled tight junctions must occur, and acidic pH triggers the dissociation of cancer cells [33,34]. With regard to the metastatic process in several cancer systems, acidic pH was shown to induce activation of acid-sensing ion channel (ASIC) with intracellular Ca^2+^ ([Ca^2+^]_i_) increases to activate EMT in pancreatic cancer cells [35]. Cancer cells need enzymes such as metalloproteinase (MMP) [36] and cathepsin [37] to penetrate tissue barriers for invasion. The enzymes secreted from cancer cells are activated by the sodium hydrogen exchanger (NHE)-induced acidification of the extracellular environment [38]. In addition, adaptation to the acidic pH triggers melanoma cell invasion [39,40,41]. Hwang et al. demonstrated that cancer cell migration was regulated by extracellular modulation through bicarbonate transporters, including anion exchanger 2 (AE2) and sodium bicarbonate cotransporter-n1 (NBCn1) [42,43]. Bicarbonate plays a role in intracellular pH maintenance to regulate physiological functions [44]. The activation of AE2 or NBCn1 transports bicarbonate ions, and the electrolyte flux was reported to induce A549 lung cancer cell migration [42,43]. Thus, adjustment of the extracellular pH is considered a critical strategy for treating cancer.

PyK2 has been associated with pH alterations. Li et al. demonstrated that PyK2 was a pH sensor and activator in the kidney [45]. The phosphorylation of PyK2, which is located on the basolateral side of renal epithelial cells, rapidly occurred in an acidic medium [45]. Activated PyK2 stimulated NHE3, followed by the release of H^+^ to acidify the luminal side of the renal epithelial cells [45]. No et al. demonstrated the precise mechanism of PyK2-induced NHE3 activation. In intestinal brush border cells, lysophosphatidic acid-induced epidermal growth factor receptor (EGFR) activation phosphorylated PyK2, and sequentially, p90 ribosomal S6 kinase (RSK) was phosphorylated through the involvement of PyK2 [46]. Phosphorylated RSK phosphorylated NHE3 to traffic NHE3 to the apical membrane of the intestine [46]. PyK2 increased ion movement through sodium-coupled dicarboxylate transporter-1 (NaDC-1) and H^+^-ATPase. NaDC-1 transported 3Na^+^ with citrate^3−^, which is used in the citric acid cycle [47,48]. Citrate plays important roles in the kidney, including the disruption of kidney stone formation [49,50], and is the principal base of urine [51]. Acidic media activated NaDC-1 in renal proximal tubule cells to uptake citrate [52]. The inhibition of PyK2 through a dominant-negative mutation decreased citrate uptake, and the deletion of PyK2 attenuated mouse blood citrate levels [52]. Reduced citrate levels are associated with potential kidney stone formation, which is affected by changes in the acid-base balance [52]. In addition, cellular adaptation to acidic pH was mediated by PyK2-associated H^+^-ATPase through the phosphorylation of extracellular signal-regulated kinase (ERK) 1/2 in mouse-derived outer medullary collecting ductal cells [53]. PyK2 is activated by ion channel ASIC1a. The acidosis of osteoclasts activated ASIC1a and subsequently increased the influx of Ca^2+^ [54]. The increased Ca^2+^ phosphorylated PyK2 and Src to up-regulate integrin, which induced cellular adhesion and migration [54]. Therefore, PyK2 triggers the acidification of extracellular pH and is modulated by extracellular pH. Thus, in this paper, we focused on the relationship between cancer (especially metastasis) and PyK2 with regard to pH alterations and the related mechanisms.

## 2. The Effect of PyK2 on Cancer Progression

The cancer environment is acidic, and this acidic microenvironment provides favorable conditions for cancer cell migration or invasion [31]. PyK2 is overexpressed in numerous cancers, including non-small lung cancer [55], breast cancer [56], colorectal cancer [57], and liver cancer [58]. PyK2 overexpression has a regulatory role in cancer tumorigenesis, including cell proliferation, migration, invasion, and metastasis. PyK2 activation is associated with the initiation of olfactory receptor signaling in prostate cancer cells [59], pre-malignant signaling in pancreatic ductal adenocarcinoma [60], and pituitary adenylate cyclase-induced phosphorylation-activating polypeptide receptors in non-small lung cancer cells [61]. In this section, we focused on the role of PyK2 as a signaling molecule in cellular metabolism, including proliferation, migration, and invasion, and highlighted the role of PyK2 as a therapeutic target in various cancers.

### 2.1. Role of PyK2 in Tumorigenesis and Proliferation

PyK2 is associated with the proliferation of cells such as fibroblasts, smooth muscle cells, and osteoblasts [62,63,64]. The knockdown of PyK2 decreased mouse embryo fibroblast growth, and deletion of the PyK2 gene attenuated the proliferation of megakaryocyte-induced osteoblasts in mice [62,64]. Platelet-derived growth factor (PDGF), which induces cellular proliferation, was shown to increase the phosphorylation of PyK2 in vascular smooth muscle cells [63]. In addition to fibroblasts and smooth muscle cells, the phosphorylated pY402 and pY881 forms of PyK2 are up-regulated in non-small cell lung cancer (NSCLC) tissues. However, the pY881 form was associated with different survival rates in patients with NSCLC [55]. Patients with a low expression of PyK2 (pY881) survived longer than patients with a high expression of PyK2 (pY881) [55]. Thus, the development of NSCLC is mainly regulated by the pY881 form of PyK2. PyK2 was also reported to phosphorylate the Y216 site of GSK3β to promote Wnt/β-catenin pathway signaling [57]. Wnt signaling is a major pathway in developing colorectal cancer [65]. Wnt signaling is activated by the inactivation of adenomatous polyposis coli (APC), which is a tumor suppressor, and the activation of β-catenin, which is a proto-oncogene [66]. The inactivation of APC resulted in the GSK3-induced phosphorylation of β-catenin [67]. The inhibition of PyK2 kinase activity attenuated adenoma formation in mice with APC inactivation [57]. Eph receptor 2 (EphA2), which is a tyrosine kinase, has been studied as a tumor suppressor [68]. Knocking down EphA2 induced skin cancer and ERK phosphorylation [69]. The tumorigenesis of cholangiocarcinoma was enhanced by EphA2 activation with the activation of PyK2 [70]. However, the relationship between PyK2 and cancer proliferation in prostate cancer is regulated by PyK2 expression, regardless of phosphorylation. PyK2 expression is up-regulated in prostate cancer cells and correlated with the enhanced expression of androgen receptors [71]. The inhibition of PyK2 expression attenuated the growth of prostate cancer cells and down-regulated androgen receptor expression and activity [71]. However, the overexpression of PyK2 and phosphorylation of the androgen receptor increased the growth of prostate cancer cells [71].

### 2.2. Migration, Invasion, and Metastasis

Several studies of PyK2 have verified that the over-expression and activation of PyK2 are related to cancer metastasis in numerous cancer cells, such as breast cancer, liver cancer, pancreatic cancer, prostate cancer, and glioma [19,58,72,73,74,75]. Not only does PyK2 mainly act as an up-regulated protein, but also the expression of PyK2 acts as an oncogenic protein for metastatic cancer. Cancer metastasis is initiated by EMT, which induces mobility to transform the shapes of cancer cells [76]. PyK2 promotes EMT or the migratory properties of various cancer cells. Briefly, PyK2 overexpressed in non-metastatic hepatocellular carcinoma (Hep3B) gave rise to EMT characteristics, which included enhanced membrane ruffle formation and the down-regulation of the cell adhesion molecule E-cadherin and the mechanical stress-associated protein cytokeratin [77]. In contrast, the knockdown of PyK2 modulated the morphology of BT-549 breast cancer cells to epithelial-like cells with the enhanced expression of E-cadherin [78]. The expression of PyK2 was increased by treating the epidermal growth factor (EGF) and transforming growth factor-β (TGF- β), which triggered EMT in MDA-MB-231 cells [78]. In high-grade breast cancer tissue, which manifests metastatic features, PyK2 expression was higher than in low-grade breast cancer tissue [78]. The migration of ovarian cancer and glioma cells was also stimulated by the up-regulation of PyK2. Chemokine ligand 18 (CCL18), which is a breast cancer cell migration stimulatory factor, enhanced the activation of PyK2 in ovarian cancer cells (CaOV3 and OVCAR3) [79]. The overexpression of PyK2 increased the migration of breast cancer cells, whereas the knockdown of PyK2 decreased breast cancer cell migration [79]. In addition, CCL18-induced increases in breast cancer cell (MDA-MB-231) migration, accompanied by the activation of PyK2 and Src [80], whereas CCL18-induced cell migration was attenuated by siRNA-PyK2 and siRNA-Src [80]. The overexpression of PyK2 enhanced glioma-cell (SF767 and G112) migration [81] and PyK2 siRNA-attenuated glioma-cell (A172, U87, HS683, and C6) migration [82]. Mutation of the FERM domain of PyK2 decreased the migration of glioma cells [83], suggesting that the FERM domain of PyK2 is involved in cellular migration.

**Figure 2:**
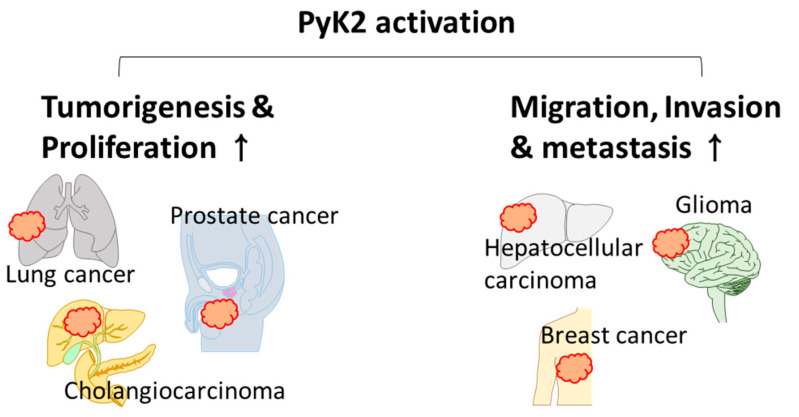
Schematic illustration of the effect of PyK2 on various cancers. PyK2 activation enhances tumorigenesis, cancer proliferation, migration, invasion, and metastasis. The affected cancers are lung cancer, prostate cancer, cholangiocarcinoma, hepatocellular carcinoma, glioma, and breast cancer.

PyK2-mediated invasion is evaluated using the Matrigel-coated Transwell assay. The application of siRNA-PyK2 decreased the invasion of A549 lung cancer cells [84], and the down-regulation of tropomyosin-related kinase B attenuated PyK2 phosphorylation (Y402) and subsequently decreased A549 migration [84]. The growth factors, EGF and heregulin (HRG), enhanced the invasion of breast cancer cells (MCF7, T47D, and SKBR3), accompanied by an increase in PyK2 phosphorylation [85]. The knockdown of PyK2 attenuated breast cancer cell migration via the down-regulation of MMP9, which degrades the ECM to penetrate the blood vessel barrier [85]. In non-cancer systems, the inhibition of PyK2 by the PyK2 inhibitor PF-4594755 decreased the migration of primary cultured mouse smooth muscle cells without a decrease in proliferation [86]. PyK2 regulates the migration of immune cells, including cytotoxic T lymphocytes (CTLs) and macrophages [87,88]. PyK2 Inhibition by PF-431396 decreased the migration of primary cultured mice CTLs [87] and attenuated the hydrogen sulfide-stimulated migration of RAW264.7 cells [88]. PyK2 overexpression stimulated the migration of mouse cortical neurons [89]. Thus, PyK2 plays a critical role in cell progression and migration (Figure 2), and strategies for regulating PyK2 might provide a new therapeutic approach against cancer.

## 3. PyK2-Associated Molecules in Cancer

The acidic milieu is a favorable condition in cancer systems. Various evidence has shown that the activation of PyK2 regulated cancer progression and migration. Thus, in this chapter, we summarized the mechanism of molecular interaction in regulating PyK2 activity in cancer and PyK2-associated strategies against cancer.

### 3.1. Chemical Reagents

Kinase inhibitors, which decrease the phosphorylation of PyK2, suppress cancer viability and migration. Mitoxantrone, which targets the ATP-binding site of FAK and decreases the auto-phosphorylation of FAK, decreased PyK2 kinase activity in BT474 breast carcinoma cells [90]. Moreover, the tyrosine kinase inhibitor, SAR103168, decreased PyK2 phosphorylation by the downstream inhibition of Src in human myeloid leukemia cells (KG1) [91]. SKI-606, which is an Src inhibitor, decreased the phosphorylation of PyK2 and the migration and invasion of MDA-MB-468 breast cancer cells without affecting proliferation, suggesting that PyK2 induced the migration of breast cancer cells by activating Src [92]. The reactive oxygen species (ROS) inducer eicosapentaenoic acid (EPA), which dephosphorylates PyK2, exhibited anti-cancer effects by decreasing the proliferation and migration of PC3 prostate cancer cells [93]. PyK2 regulation ameliorated drug resistance to cisplatin and doxorubicin. The overexpression of PyK2 increased the effect of cisplatin in human hepatocellular carcinoma cells to decrease proliferation [94]. Alpha-naphthoflavone (ANF) decreased the phosphorylation of PyK2 in MCF-7 cells, and the combination of doxorubicin and ANF reduced breast cancer volume compared with a single treatment of doxorubicin or ANF in breast cancer-xenografted mice [95].

### 3.2. Interaction of Protein with PyK2 in Cancers

PyK2 interacts with various proteins, and its interactions with PyK2 have been developed in cancer systems. For example, the Csk homologous kinase (CHK), which inhibits the activation of Src family kinases, physically binds to PyK2 in T47D breast cancer cells [96]. A deficiency of heat shock cognate protein 70 (hsc70), which promotes the proliferation and migration of human glioma cells (U251 and U87), attenuated the phosphorylation of Src, FAK, and PyK2 [97]. Rb1-inducible coiled-coil 1 (RB1CC1) is a tumor suppressor that is considered to be a therapeutic target in renal carcinoma [98]. The overexpression of RB1CC1 decreased the phosphorylation of PyK2 and doxorubicin, which increased RB1CC1 expression and reduced the size of xenografted renal cell carcinoma tumors [99]. A decrease in PyK2 phosphorylation decreased cancer progression, and cancer migration and invasion were affected by PyK2 and its interactive proteins. Melatonin exerted an anti-cancer effect on brain tumor cells [100], and treatment with melatonin reduced the phosphorylation of PyK2 and the expression of alpha V beta 3 (αVβ3) integrin in U251 glioma cells [101]. The knockdown of αVβ3 decreased PyK2 phosphorylation and the migration of U251 cells [101].

### 3.3. Chemokine-Related Molecules

PyK2 is regulated by chemokine-related proteins, including the C-C motif chemokine ligand/receptor (CCL/CCR) and C-X-C motif chemokine ligand/receptor (CXCL/CXCR). CCL and CXCL recruit monocytes and neutrophils to the tumor site [102,103,104]. Thus CCL- and CXCL-related immune pathways have a close connection with cancer therapy. For example, CCL2 and CCL5, which are secreted by mesenchymal stem cells, induced PyK2-dependent chemoresistance in ovarian cancer cells (Skov3 and Ovcar3) [105]. CCL2- and CCL5-mediated chemoresistance was decreased through treatment with the PyK2 inhibitor PF-431396 [105]. PyK2 also plays a role in tumor viability and reactions with CCL2 and CCL5. ADP-ribosylation factor-GTPase activating protein (Arf-GAP), with an SH3 domain, ankyrin repeat, and PH domain-containing protein 1 (ASAP1, also called DDEF1 or AMAP1), is highly expressed on breast cancer cells and mediates breast cancer invasion and metastasis [106]. Treatment with CCL18 increased ASAP1 phosphorylation, and the knockdown of PyK2 prevented CCL18-induced increases in p-ASAP1 in MCF-7 cells [107]. p-ASAP1 trans-locates toward the plasma membrane to form a complex with PyK2 in the presence of CCL18 [107]. Treatment with CCL18 stimulated cellular adhesion, migration, and invasion, whereas the inhibition of ASAP1 through siRNA attenuated CCL18-induced cellular mobility features in MCF-7 cells [107]. CCR7 also plays a role in cancer migration and invasion. CCR7, which binds with CCL19, stimulated the phosphorylation of Janus kinase 2 (JAK2) and signal transducer and activator of transcription 3 (STAT3) in head and neck squamous cell carcinoma cell lines (PCI-4B and PCI-37B) [108]. The phosphorylation of JAK2 and STAT3 was attenuated by the PyK2 inhibitor A9 in PCI-4B and PCI-37B cells [108]. The inhibition of JAK2 and STAT3 decreased the migration and invasion of PCI-4B and PCI-37B cells [108], and treatment with CXCL12 induced the chemotaxis and chemoinvasion of MDA-MB-231 cells [109]. CXCL12, which binds with CXCR4, induced PyK2 phosphorylation in breast cancer cells (MDA-MB-231) [109]. The tyrosine phosphatase inhibitors vanadate and phenylarsine oxide attenuated the chemotaxis and chemo-invasion of MDA-MB-231 cells [109]. Although accumulating evidence has been reported, further verification of multiple chemokine/PyK2-associated mechanisms will provide potential strategies for treating cancer.

### 3.4. Ca^2+^ Channels and Transporters

PyK2 phosphorylation is also modulated by the signaling messenger, intracellular Ca^2+^. PyK2 senses Ca^2+^ signaling through calmodulin (CaM), and PyK2 has a CaM-binding motif [110]. In hypoxia, increases in the intracellular Ca^2+^ concentration ([Ca^2+^]_i_) induced PyK2 phosphorylation [111]. Treatment with the Ca^2+^ chelator BAPTA attenuated hydrogen peroxide (H_2_O_2_)-stimulated PyK2 phosphorylation [112]. Ca^2+^ signaling plays important roles in muscle contraction, neurotransmitter release, immune cell differentiation, fluid secretion, and cell proliferation [113,114,115,116]. Cancer progression and cancer cell death are especially affected by Ca^2+^ signaling [117,118,119,120,121,122,123]. In addition, the activation of Ca^2+^ channels and transporters regulates the interaction between PyK2 and cancer activity. [Ca^2+^]_i_ is increased by the activation of various Ca^2+^ channels and transporters that are located on intracellular organelle and plasma membranes. Intracellular Ca^2+^ is stored in intracellular organelles, including the nucleus, mitochondria, and endoplasmic reticulum (ER), to maintain Ca^2+^ homeostasis. The mitochondrial protein Lon is involved in protein quality control and maintains mitochondrial homeostasis [124,125]. The overexpression of Lon induced the phosphorylation of PyK2, increased [Ca^2+^]_i_ through the involvement of a mitochondrial Na^2+^/Ca^2+^ exchanger, and enhanced chemoresistance to cisplatin in human oral squamous carcinoma cells (OEC-M1) [126].

The ER, another intracellular Ca^2+^ store, contains a Ca^2+^ sensor protein called stromal interaction molecule 1 (STIM1) [127]. This Ca^2+^ sensor STIM1 recognizes depletions in ER Ca^2+^ by a STIM1-Orai1 complex on plasma membranes and mediates increases in [Ca^2+^]_i_ in a process called store-operated Ca^2+^ (SOC) entry (SOCE) [127]. The down-regulation of STIM1 decreased the EGF-induced phosphorylation of PyK2 and enhanced the focal adhesion of cervical cancer cells (SiHa) [128]. The knockdown of STIM1 inhibited tumor progression in a cervical cancer mouse model [128]. Additionally, the inhibition of SOCE by the SOCE inhibitors shOrai1 and SKF96365 increased PyK2 dephosphorylation and focal adhesion in mouse glioma cells (C6), human glioma cells (U251 and SNB19), and human melanoma cells (WM793) [129,130,131]. Transient receptor potential melastatin 2 (TRPM2), which is located on plasma membranes, inhibited the effect of the anti-cancer drug doxorubicin in neuroblastoma [132]. The knockdown of TRPM2 enhanced the anti-cancer effects of doxorubicin to decrease PyK2 phosphorylation. Hirschler-Laszkiewicz et al. suggested the inhibition of TRPM2 as a target for cancer therapy in patients with doxorubicin chemoresistance [132]. Although the effect of modulating TRPM2 channels must be carefully verified because of conflicting views of TRPM2 (Ca^2+^ influx through TRPM2 induces apoptosis through goldnano-conjugated doxorubicin) [133], enhanced PyK2 phosphorylation through Ca^2+^ signaling presents further challenges in verifying the precise mechanism for cancer therapy.

### 3.5. Reactive Oxygen Species

In cancer cells, oxidative modification has pathological roles in protein alterations through the involvement of second messengers, including ROS, H_2_O_2_, reactive nitrogen species (RNS), and nitric oxide (NO) [134,135,136]. Oxidative stress has been considered a hallmark of cancer to increase cancer progression, including proliferation and invasion [137,138]. Oxidative stress also affects PyK2 activation in cancer cells. Treatment with estrogen produced ROS, and increased PyK2 phosphorylation in human breast cancer cells, including MCF-7, T47D, ZR75-1, and MDA-MB-468 cells [139]. Hypoxic conditions increased the phosphorylation of PyK2 in U251 glioma cells [101]. The migration and invasion of U251 cells were increased by hypoxic stimulation, and the knockdown of PyK2 inhibited hypoxia-induced U251 cell migration [101].

**Figure 3:**
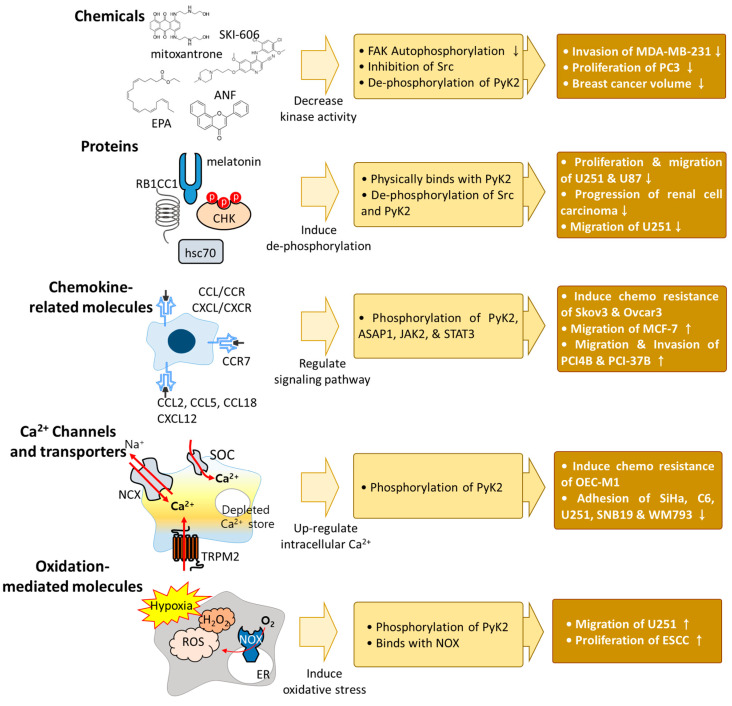
Schematic illustration of PyK2-associated molecules. Various molecules affect PyK2 activation, including chemical reagents, interactive proteins, chemokine-related molecules, Ca^2+^ channels, transporters, and oxidation-mediated molecules. The phosphorylation of PyK2 induces cancer cell migration and proliferation. Various effector signals and chemicals exert different phosphorylation effects on PyK2. Thus, verification of the phosphorylation status of PyK2 could be a prognostic marker for evaluating cancer progression.

PyK2 was reported to bind with dihydronicotinamide adenine dinucleotide phosphate (NADPH) oxidase (NOX) in KySE30 and KySE410 esophageal squamous cell carcinoma (ESCC) [140]. Hypoxia induced the phosphorylation of PyK2 and the production of H_2_O_2_ in ESCC [140]. NOX5 shRNA and PyK2 mutation decreased H_2_O_2_ levels in ESCC cells under hypoxic conditions and decreased ESCC proliferation [140]. Oxidation also plays a critical role in cardiovascular functions and CTLs [112,141,142,143,144]. Treatment with H_2_O_2_ enhanced the phosphorylation of PyK2 in mouse left ventricular myocytes [112] and H9c2 cardiomyocytes [142]. The deletion of PyK2 attenuated the production of NO in primary cultured-mouse endothelial cells from the aorta [141]. Additionally, treatment with H_2_O_2_ stimulated PyK2 phosphorylation, and the activation of PyK2 phosphorylation increased the production of ROS in CTLs [143]. Overall, oxidative stress induces PyK2 phosphorylation with tumor progression. Thus, the development of antioxidants and modulation of PyK2 phosphorylation provide potential strategies for cancer treatment. The mechanism of the various molecules involved in regulating PyK2 activity in cancer systems is shown in Figure 3.

## 4. Future Perspectives

PyK2 has been studied as a key regulator of cancerous processes. pH-associated kinase PyK2 is regulated by various molecules such as chemical reagents and interactive proteins, including chemokine-related and Ca^2+^-related molecules, as well as oxidation-related molecules, in cancer cells. The features of the acidic microenvironment and Ca^2+^ signaling in PyK2-associated molecular mechanisms have been demonstrated. Thus, further investigation is required to include its multiple regulators. Cells possess various ion transporters and channels, including those discussed above, and electrolyte transporters such as potassium, sodium, and chloride are also involved in cellular systems. Thus, further experimental evidence, including the relationship between ion channels and transporters and cancer, should be determined. In addition, investigations of PyK2 as an extracellular milieu-sensing protein in cancerous processes might provide further information on the responsiveness to the cancer milieu.

## Data Availability

Not applicable.
